# Ethnic and age-related fat free mass loss in older Americans: The Third National Health and Nutrition Examination Survey (NHANES III)

**DOI:** 10.1186/1471-2458-5-41

**Published:** 2005-04-19

**Authors:** Thomas O Obisesan, Muktar H Aliyu, Vernon Bond, Richard G Adams, Abimbola Akomolafe, Charles N Rotimi

**Affiliations:** 1 Section of Geriatrics, Department of Medicine, Howard University Hospital, Washington DC, USA; 2 Department of Epidemiology, University of Alabama at Birmingham, Birmingham, AL, USA; 3 Department of Physical Education, Howard University Washington DC, USA; 4 Pulmonary and Critical Care, Department of Internal Medicine, Howard University Hospital, Washington DC, USA; 5 Division of Geriatrics, Morehouse School of Medicine, Atlanta, GA, USA; 6 National Human Genome Center, Genetic Epidemiology Unit, Department of Microbiology, Howard University, Washington DC, USA

## Abstract

**Background:**

Although age-related loss of fat free mass (FFM) is well known, there is paucity of data on national estimates, and on the differential influence of ethnicity on the decline in FFM with increasing age. We determined whether age-related loss in FFM and fat free mass index (FFMI) vary by gender and or ethnicity, using representative data from the Third National Health and Nutrition Examination Survey (NHANES III).

**Methods:**

Analyses were limited to 5,803 non-institutionalized, non-Hispanic Whites and African Americans (Blacks) over the age of 40 years. Body density was calculated from the sum of 3-skinfolds, and percent body fat estimated from body density. FFM was estimated by subtracting body fat from body weight, while FFMI was defined as FFM (kilograms) divided by the square of body height (meter^2^).

**Results:**

Overall FFM and FFMI were significantly higher in black women than white women (*P *= 0.001; *P *= 0.001 respectively), but similar in black men compared to white men. Age-related decline in FFM reached significance level earlier in black men (at age 65–69) than white men (at age 70–74), and in black women (at age 70–74) than white women (at age 75–79). Similar decline in FFMI was noted in men and in women. In multivariate analyses, FFM significantly associated with ethnicity (*p *= 0.012) and with age (*p *< 0.001) in women, but only with age (*p *< 0.001) in men. In men and in women, FFMI significantly associated with ethnicity (*p *< 0.001; *p *= 0.003 respectively) and with age (*p *< 0.001; *p *= 0.004 respectively).

**Conclusion:**

Age-related loss and decline in FFM and FFMI in older Americans is higher for black men and women, than for white men and women. The development of focused population-based preventive strategies is likely to improve functional independence in the aged.

## Background

Advancing age is associated with a number of changes in body composition. Notable among these changes is the reduction in FFM, that occurs primarily as a result of losses in skeletal muscle mass [1], a condition referred to as sarcopenia. For example, older men aged 80 years or older have 25 percent less leg muscle mass than young men between the ages of 20 and 29[2]. FFM has been closely linked with reduction in muscle strength in the aged. Further, a greater reduction in both muscle mass and strength have been reported in older nursing residents with history of falls. Thus reduction in muscular strength is associated with increased risk of falls and hip fracture, and prevalence of disabilities[3]. Independent of fall risk, low FFM has been linked to higher rates of all-cause mortality in women in the United States[4]. Therefore, losses in FFM with advancing age have important health implications.

Higher FFM and lean mass to height ratio in Blacks than Whites have been reported [5], but mostly in young non-representative samples. There is limited information on national estimates, and on the existence of differential influence of ethnicity on the decline in FFM with increasing age. Given the inverse association of FFM with all cause mortality[6] and the reported higher FFM in Blacks, lower mortality rates in Blacks would be expected. To the converse, both all cause mortality[7] and FFM are know to be higher in Blacks, suggesting that the relationship of FFM with ethnicity and all cause mortality may be due to reasons other than differences in absolute FFM. We hypothesized that black men and women will have higher degree of age-related decline in FFM and FFMI despite higher mean values in Blacks, using data from NHANES III. The characterization of changes in FFM across several decades of life is crucial to our ability to reduce the burden of sarcopenia, osteoporosis, falls and hip fracture, as well as associated mortality.

## Methods

In order to characterize FFM in older populations in the U.S., we analyzed data from NHANES III. The data for NHANES III were collected by the National Center for Health Statistics of the Centers for Disease Control and Prevention between 1988 and 1994, using a national multistage, probability sample of the civilian non-institutionalized population of the United States. The initial NHANES III sample consisted of 33,994 individuals, 2 months to 99 years of age [8]. The survey design ensured over-sampling of Blacks, and those 60 years and older. Data were collected from each participant through face-to-face interview, physical examination, and laboratory analyses. The overall survey design and provisions for informed consent have been previously detailed [8].

Subjects in the present study included 8,679 non-institutionalized Whites and Blacks over the age of 40 years. Of this number, complete data on anthropometric measurements were only available for 5,803 persons. Age was categorized as decades of life up to the age of 79 years, while persons ages 80 and older were separately grouped to avoid over-stratification and loss of statistical power. We also categorized age by increments of 5 years for the purpose of graphical representations only.

### Anthropometric measurements

Weight and standing height were determined according to standardized procedures [9]. Weight was recorded to the nearest 0.01 kg, while height was recorded to the nearest 0.1 cm. Body mass index (BMI) was calculated as weight in kg divided by height in meter squared (m^2^). Except for a few measurements that were taken on the left side of the body because of examinees' limitations, the rest of the anthropometric measurements were taken on the right side of the body using standard procedures and were recorded to the nearest 0.1 mm [9]. All skinfolds were measured using Holtain skinfold calipers on the right side of the body. With the examinee standing erect and the arms hanging freely at the sides, the triceps skinfold was measured on the posterior surface at the midpoint of the right upper arm. The suprailiac skinfold was measured at a 45-degree angle at the marked iliac crest. Measurement of the thigh skinfold was taken from a vertical line over the quadriceps muscle at midline of the thigh, and half way between the top of the patella and the inguinal crease [9]. All examiners were trained in standard procedures for obtaining measurements. Out-of-range measurements were verified to differentiate errors in measurement from high values for very small or very large examinees.

Because relevant anthropometric measurements were available only for the triceps, suprailium and thigh, body density was estimated from the sum of 3 skinfolds, (triceps, suprailium, and thigh) and age, based on the equation of Jackson and Pollock (body density = 1.0994921-0.0009929 * (triceps + suprailium + thigh) + 0.0000023 * (triceps + suprailium + thigh)**2 - 0.0001392 * (age)) [10], and percent body fat was estimated from the Brozek equation (percent body fat=((4.570/body density) - 4.142) * 100) [11]. FFM was calculated by subtracting body fat from body weight. Based on the analogy of BMI and the high correlation between FFM and height, FFMI was derived by dividing FFM (kg) by the square of height (m^2^)[12].

### Statistical analyses

Because our primary interest was in how ethnicity influenced age-related differences or decline in FFM by gender, initial analyses were conducted for each of the 4 demographic subgroups formed by the combination of sex and ethnicity. Separate analyses were conducted for each decade of life beginning from the 4^th ^to the 8^th ^decade. Mean values were calculated for FFM and FFMI. The χ^2 ^and t-test statistics and 95 percent Confidence Interval were used to determine statistical significance. A General Linear Regression Model for continuous variable response, using the method of weighted least squares adapted to survey data [13] was used to test the association between the dependent variable (FFM) and independent variables (age, ethnicity and gender) each in a bivariate model. Similar models were constructed for FFMI as a second dependent variable. Estimates of individual regression coefficients (the vector of beta values) and standard errors were computed. Because of our interest in how ethnicity influenced age-related changes in FFM in men and in women, we tested for "ethnicity*gender" interaction (dependent variable = ethnicity, gender, ethnicity*gender); and "ethnicity*age" interaction (dependent variable = ethnicity, age ethnicity*age). Because of the observed gender- and ethnicity interaction, and the differences in FFM and FFMI in the initial analyses, final multiple regression models to test for the associations of ethnicity and age, with FFM and FFMI were conducted separately for each gender. The model for FFM included adjustment for BMI. However, because FFMI already adjusted for height, the regression model for FFMI did not include height. This precaution was taken to avoid co-linearity and the bias of structural relationship between FFMI and height. All initial analyses were performed using the Statistical Analysis System (SAS) package [14]. Given the multistage complex survey design of NHANES III, final analyses were conducted in SUDAAN (Proc Descript; Proc Regression) to produce a more robust variance estimate [15]. Sample weights were included in the final analysis to account for over sampling and non-response adjustment.

## Results

### Subject characteristics

A total of 4,072 Whites (2106 men and 1966 women) and 1731 Blacks (948 men and 783 women) were included in the final analyses (Table 1). Although Blacks had overall higher body weight, standing height and BMI compared to Whites, these differences did not reach statistical significance. Among women, Blacks weighed more, were taller, and had higher body mass index than white women. In contrast, white men had higher waist-hip-ratio and triceps skinfold than black men.

**Table 1:** Anthropometric measurements and other characteristics of the study sample. The Third National Health and Nutrition Examination Survey (NHANES III 1988–94).

**Characteristics**	**Non-Hispanic Whites**		**African Americans**	
	**Men**	**Women**	**Men**	**Women**
	Mean ± SE	Mean ± SE	Mean ± SE	Mean ± SE
	(N = 2,106)	(N = 1,966)	(N = 948)	(N = 783)
Age (years)*	65.94 (14.2)	66.62 (14.5)	58.41 (12.9)	58.51 (13.6)
Body Weight (kg)*	81.20 (15.7)	68.28 (16.0)	81.40 (17.8)	78.64 (20.0)
Standing Height (cm)	174.00 (7.1)	159.54 (7.0)	174.48 (7.4)	161.91 (6.5)
Body Mass Index (kg/m^2^)	26.75 (4.5)	26.78 (5.8)	26.66 (5.2)	29.95 (7.2)
Triceps Skinfold (mm)	13.49 (5.9)	23.29 ± (8.1)*	12.34 (6.8)	26.08 (9.2)*
Suprailiac Skinfold (mm)	20.41 (8.9)	20.03 (9.6)	20.40 (10.6)	25.04 (10.3)*
Thigh Skinfold (mm)	15.09 (7.6)	30.40 (8.8)	12.48 (7.1)	29.40 (9.7)
Body Density (g/cm^3^)	1.05 (0.01)	1.03 (0.0)	1.05 (0.0)	1.03 (0.0)
Fat Free Mass (kg)	64.26 (0.3)	46.65 (0.2)*	63.87 (0.4)	49.53 (0.4)*
Fat Free Mass Index (kg/m^2^)	20.81 (0.1)	17.91 (0.1)*	20.82 (0.1)	18.82 (0.1)*

Note: Values are mean (SE); * Indicates statistical significant difference for within gender comparison of Non-Hispanic Whites with non-Hispanic African Americans at two-sided p < 0.05.

### Ethnicity-based comparison

Collectively, mean FFM and FFMI were similar between Blacks and Whites. However, when stratified by gender-ethnicity, black women had higher FFM and FFMI than White women (FFM difference: 2.88 ± 0.28 kg; *p *< 0.01) and (FFMI difference: 0.91 ± 0.10 kg/m^2^; *p *< 0.01) (Table 1). The significantly higher FFM and FFMI in black women persisted through all decades of life, except in the 8^th ^decade or higher. Overall, Blacks weighed higher than Whites (difference 5.53 ± 0.44 kg; *p *< 0.001) and this was explained by higher values in black women (difference 10.35 ± 0.59 kg; *p *< 0.001) (Table 1).

### Age-based comparison

**Figure 1:**
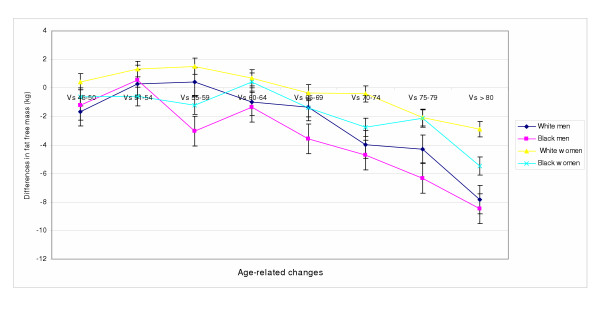
Age-related changes in fat free mass in older white and black men and women. The Third National Health and Nutrition Examination Survey (NHANES III 1988–94)

Using age group 40 – 45 as the reference, we observed exponential decline in FFM and body weight with advancing age in men and in women. This decline varied by gender and ethnicity (Figure 1). In Blacks and Whites combined, both FFM and FFMI peaked earlier in men (at age 51–54 yrs) than in women (at age 55–59 yrs). In black men, the decline in FFM started about 5 years earlier (at age 51–54 years) compared to white men (at age 55–59 years), and reached significance level at age 65–69 in black men and age 70–74 in white men.

Although FFM peaked earlier in white women compared to black women, statistically significant decline was reached earlier in black women at age 70–74 (*p *= 0.025) compared to age 75–79 in white women (*p *= 0.01). Initial onset of decline in FFM occurred almost a decade after that of men of similar ethnic background. In both white and black women, decline in FFMI was also noted, but did not attain significance level. Furthermore, decline in FFMI was noted in black men starting at age 60–64 and reaching significance at age 70–74 (*p *= 0.047). In white men, decline started at age 70–74, and reached significant level in the eight decade (of life (*p *< 0.01).

### Multiple regression analyses

In multivariate analysis for the entire group including ethnicity, age and gender, while adjusting for BMI, FFM significantly associated with ethnicity (*p *< 0.043), age (*p *< 0.001), and gender (*p *< 0.001). In separate analyses for men and for women, FFM significantly associated with ethnicity (*p *< 0.012) and age (*p *< 0.001) in women, but only with age (*p *< 0.001) in men (Table 3). This confirmed our preliminary observation of higher FFM in black women compared to white women, and significant age-related changes in FFM. However, black and white men were similar in FFM. Because FFMI was already adjusted for height, the multivariate model for FFMI was adjusted for body weight instead of BMI. The association of FFMI with ethnicity, age and gender in the entire group was similar to that observed for FFM (all *p *< 0.001). In men and in women, FFMI significantly associated with ethnicity (*p *< 0.001; *p *= 0.003 respectively) and with age (*p *< 0.001; *p *= 0.004 respectively) (Table 3).

**Table 3:** Regression analysis of fat free mass, and fat free mass index by age in white, and black men and women. The Third National Health and Nutrition Examination Survey (NHANES III 1988–94).

		**Men**			**Women**		
	Characteristics	Beta Coefficient	Standard Error	*P *Value	Beta Coefficient	Standard Error	*P *Value
FFM (kg)							
	Ethnicity	-0.338	0.276	0.227	-0.821	0.313	0.012
	Age (yrs)	-0.109	0.011	<0.001	-0.125	0.021	<0.001
	BMI (kg/m^2^)	1.527	0.039	0.001	1.053	0.032	<0.001
FFMI (kg/m^2^)							
	Ethnicity	-0.333	0.070	<0.001	-0.456	0.147	0.003
	Age (yrs)	0.017	0.003	<0.001	-0.015	0.005	0.004
	Body Weight (kg)	0.122	0.003	<0.001	0.479	0.006	<0.001

**Note: **Statistical significance was set at *P *≤ 0.05; Fat free mass model adjusted for body mass index; and fat free mass index model adjusted for body weight. FFM indicates fat free mass; FFMI indicates fat free mass index; and BMI indicates body mass index.

## Discussion

Based on a representative national epidemiological data, we observed significantly higher FFM and FFMI in black compared to white women, but not between black and white men. These differences were independent of body weight. Important differences in age-related decline in FFM and FFMI were also noted, starting earlier in black men than white men, but earlier in white women than black women despite a higher decline in black women. Despite the earlier onset of decline of FFM and FFMI in white women, the decline attained significance level earlier in black than white women. Overall, Blacks had greater decline in FFM than Whites.

Chumlea and colleagues reported higher mean FFM in black men and women compared to white men and women respectively [16]. An inverse association of FFM and FFMI with mortality has been reported [4]. It is not known whether this association is driven by differences in FFM and FFMI, or by dissimilarities in the onset of decline of FFM and FFMI with increasing age. Because mortality rates were reportedly higher for black men than for white men, and for black women than white women, [7] mean differences in FFM and FFMI are unlikely to significantly contribute to FFM-related mortality rates in Blacks. Although several causative factors are likely to be contributory to mortality rates in different populations, it is also possible that age-related changes in FFM and FFMI are important factors that must be considered in future studies on mortality and aging. Since clinical interventions to attenuate age-dependent decline in FFM are now available, longitudinal studies to examine the influence of age-related decline in FFM and FFMI on mortality patterns is imperative.

Because of the availability of more sophisticated measures of body composition, there has been a great deal of debate on the clinical utility of anthropometric measurements. Our findings of ethnicity-related differences in FFM in this study, is remarkably similar to recent estimates of FFM by Chumlea et al who used bioelectrical impedance (BIA) data from NHANES III [16]. Chumlea and colleagues provided the first national estimates of body composition as reference, to compare other studies. In fact, the age specific estimates of FFM in the Chumlea's study are concordant with our anthropometric estimates of FFM for white, and for black men and women. The BIA estimates of FFM, together with our anthropometric-derived FFM from the NHANES III, are analogous to DEXA-derived estimates of FFM by Gallagher et al [17]. The close approximation of our estimates with data from studies that used DEXA to estimate FFM supports the use of a generalized equation from anthropometric measurements to estimate FFM in a large representative sample, such as NHANES III, especially when adjusted for age. Despite the known methodological limitations associated with the use of generalized equations, our study provides valuable data on national estimates of FFM against which other future estimates from more robust measures can be compared.

### Ethnicity-based differences

Men of both ethnic groups were similar in mean FFM and FFMI, whereas, black women had higher body weight, FFM and FFMI than white women. Marshall et al and others previously reported higher muscle mass in Blacks compared to Whites [18,19]. Comparable to our observation of -2.88 kg mean difference in FFM between white women and black women in this study, Gallagher et al reported -2.22 kg difference in FFM between these 2 groups of women [17]. Also, Gasperino et al observed greater appendicular muscle mass in black women relative to white women [20]. While black women in our study were significantly younger than white women, the presence of differences in FFM that were maintained across several decades of life, argue against differences in age as a likely explanation for the higher FFM in black women. Though Blacks in the study were slightly heavier, and anthropometric measurements have been reported to over-estimate body density at extremes of body fatness, we found no statistically significant difference in mean BMI (a measure of body fatness) between white and black women. This suggests that bias from differences in body fatness may not explain the observed differences in FFM between the two groups. Additionally, the final multiple regression model confirmed the association of FFMI with ethnicity in men and in women, and of FFM with ethnicity in women only (Table 3).

Although, knowledge of the exact mechanisms leading to ethnic differences in muscle mass remains rudimentary, differences in androgenic activity have been suggested to play an important role. For example, Dowling and Pi-Sunyer reported higher levels of testosterone and sex hormone binding globulin in obese black women compared to obese white women [21]. Moreover, a 3- to 4-fold higher allele frequency in the human myostatin gene (GDF8 – a gene which codes for muscle mass) was reported in Blacks compared to Whites [22]. Higher FFM and FFMI in black women compared to white women despite lower levels of physical activity (a major determinant of FFM) lend additional support to the presence of important contributing factors at the molecular level.

### Age-related changes

FFM declined with advancing age starting earlier in black men compared to white men, but earlier in white women than black women (Figures 1). Using age as a continuous variable, Regression analysis showed significant decline in FFM and FFMI with increasing age in women (P < 0.001; P = 0.004 respectively) after accounting for the influence of body weight and ethnicity (Table 3). In a similarly adjusted model for men, decline in FFM and FFMI were associated with escalating age (P < 0.001; P < 0.001 respectively). This age-related decline in FFM and FFMI were greater for black compared to white women. In men, only FFMI (P < 0.001) but not FFM (P = 0.227) showed a greater decline in black men than white men. Although findings similar to some of our current observations have been reported, as far as we know, we are first to report ethnicity-related differential decline in FFM and FFMI with advancing age in the same study, using anthropometric measurements from a nationally representative sample (NHANES III). While our estimates of FFM for white men below age 70 are in agreement with reports from the Fels study by Guo and colleagues[23], our estimates for women were slightly higher, but within reported margins of error. Further, the onset of age-related decline in FFM at about age 50 in white men and women in our study are in concordance with the Fels study. The earlier onset of decline in FFM in black men is particularly interesting, given the similarity in mean body weight, standing height, BMI, suprailiac skinfold and body density to that of white men (Table 1). Similarly, white and black women did not differ significantly with respect to mean standing height, thigh skinfold and body density. Of note, is the observation of differential onset and decline in FFM and FFMI in this study that appears to parallel mortality patterns for the United States [7]. While underlying disease processes could potentially explain this observation, it is also possible that age-related decline in FFM and FFMI compared to absolute values may have more important implications for health outcomes. Moreover, low levels of physical activity in Blacks [24] may accelerate the process of sarcopenia and unmask several age-related sub-clinical illnesses. Alternatively, differential reduction in androgen and testosterone, reduction in responsiveness to trophic hormones [3], growth hormone [25], and insulin-like growth factors [3] with escalating age may play important roles. The relative increase in FFMI in white women is perhaps related to increased risk of osteoporosis-related compression fracture, reduction in standing height, and relative increase in FFMI. Independent reports from Schutz et al, and Hughes et al lend support to the observed age-related changes in FFMI in white women [26,27].

While decline in FFM in women mirrored changes in body weight, we found no consistent pattern of differential weight decline between white men and black men. The finding in women is particularly remarkable, since heavier body weight requires higher FFM for support (Table 2). Nonetheless, a greater reduction in body weight than can be explained by losses in FFM might suggest a greater reduction in absolute body fat with increasing age. Although it is possible that FFMI may overcompensate for standing height, the lack of significant correlation between FFMI and standing height in this sample makes this unlikely.

**Table 2:** Distributions of fat free mass, fat free mass index and body weight in white and black men, and in women. The Third National Health and Nutrition Examination Survey (NHANES III 1988–94).

	**Age Groups (yrs)**	**Sample Size**	**Whites Mean ± SE**	**African Americans Mean ± SE**	***P *Value**
					
**FFMI (kg/m^2^**)					
**Men**	40–49	708	20.64 ± 0.12	20.91 ± 0.14	0.153
	50–59	573	21.13 ± 0.11	20.92 ± 0.15	0.265
	60–69	718	21.00 ± 0.12	20.92 ± 0.17	0.704
	70–79	600	20.68 ± 0.14	20.33 ± 0.15	0.059
	> 80	455	19.93 ± 0.12	19.89 ± 0.32	0.904
					
**Women**	40–49	607	17.49 ± 0.10	18.70 ± 0.20	<0.001
	50–59	506	18.07 ± 0.17	18.71 ± 0.22	0.026
	60–69	581	18.09 ± 0.12	19.26 ± 0.29	<0.001
	70–79	605	18.21 ± 0.11	18.93 ± 0.25	0.014
	> 80	450	18.09 ± 0.09	18.34 ± 0.32	0.432
					
**FFM (kg)**					
**Men**	40–49	708	64.83 ± 0.52	65.30 ± 0.61	0.550
	50–59	573	65.94 ± 0.38	64.29 ± 0.59	0.012
	60–69	718	64.42 ± 0.42	63.23 ± 0.66	0.139
	70–79	600	61.50 ± 0.43	60.36 ± 0.65	0.124
	> 80	455	57.74 ± 0.46	57.23 ± 1.32	0.715
**Women**	40–49	607	46.90 ± 0.35	50.28 ± 0.66	<0.001
	50–59	506	47.66 ± 0.46	49.59 ± 0.69	0.013
	60–69	581	46.94 ± 0.40	50.15 ± 0.69	0.001
	70–79	605	45.59 ± 0.39	48.01 ± 0.62	0.002
	> 80	450	43.83 ± 0.27	45.04 ± 0.83	0.143
					
**Body Weight (kg)**					
**Men**	40–49	708	83.76 ± 1.00	82.10 ± 1.00	0.252
	50–59	573	85.35 ± 0.66	81.13 ± 1.03	<0.001
	60–69	718	83.94 ± 0.73	80.41 ± 1.13	0.013
	70–79	600	78.92 ± 0.70	76.91 ± 1.09	0.097
	> 80	455	73.25 ± 0.63	72.77 ± 2.07	0.834
					
**Women**	40–49	607	65.71 ± 0.66	71.71 ± 1.41	<0.001
	50–59	506	68.47 ± 0.93	71.83 ± 1.38	0.031
	60–69	581	66.86 ± 0.68	72.33 ± 1.09	<0.001
	70–79	605	64.11 ± 0.74	68.44 ± 1.07	0.002
	> 80	450	60.46 ± 0.45	61.93 ± 1.62	0.365

**Note: **statistical significance was set at *P *≤ 0.05; Whites and African Americans refer to respective ethnic groups of non-Hispanic origin. FFM indicates fat free mass; FFMI indicates fat free mass index; and BMI indicates body mass index.

**Figure 2:**
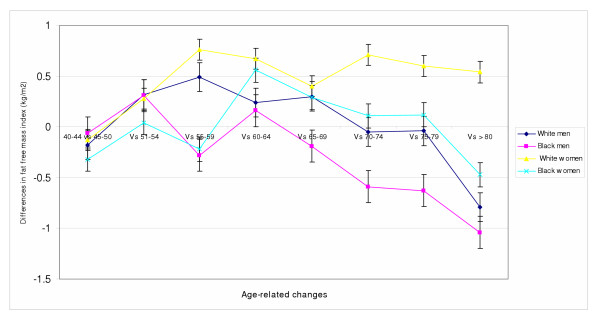
Age-related changes in fat free mass index in older white and black men and women. The Third National Health and Nutrition Examination Survey (NHANES III 1988–94)

Within each ethnic group, the decline in FFM and FFMI started earlier in men than women (Figure 1). This finding is consistent with published reports from others who showed linear decline in skeletal muscle mass with advancing age[28,29]. Unfortunately, many of the previous studies lacked the ethnic stratification, sample size and representativeness of the current study. Similar to our findings of gender-based differential decline in FFM, a Japanese study reported decreases in lean body mass in Japanese men but not in women[30]. While our current anthropometric estimates of FFM and FFMI from the NHANES III study may be at variance with estimates from a limited number of studies that showed loss of skeletal muscle mass in the 3rd decade of life[17], several other studies that used more robust methods to estimate skeletal muscle mass[31,32,23] support our current observations. Altogether, our study supports the presence of higher mean FFM in black women than white women, earlier onset of decline in FFM and FFMI in black men than white men respectively (Figures 1 and 2), and greater age-related decline in black men and women than in white men and women. Additionally, it provides support for the use of anthropometric measurements to estimate FFM in large epidemiological studies.

### Limitations

The observations from this study are subject to a number of limitations. First, at extremes of BMI and body fat, there is bias in the use of skinfold-thickness measurements to estimate body density from which percent body fat is determined. This concern is heightened in the aged because of increased centripetal fat distribution. Fortunately, Pollock's equation included adjustment for age and the final regression model in our study was additionally adjusted for BMI. While population-specific equations are appropriate alternatives and sometimes provide more accurate estimates, they are limited in generalizability. Though the equation used in the present study has greater applicability to women than men, its selection was based on the available anthropometric measurements in the NHANES III data.

Because the Pollock equation was not derived from the NHANES sample, it is possible that it may not predict body density with the same degree of precision compared to the original parent sample [33]. Further, the cross-sectional nature of the NHANES III data makes it possible that changes in FFM with advancing age may have been underestimated, given known estimates from longitudinal studies [34]. These concerns are minimized by the similarities of our findings with that of studies using Bioelectrical Impedance and DEXA to estimate FFM, and therefore, may not reduce the significance of the population-based observation from the current study. Inter-, intra-observer's and instrument bias appear unlikely in the NHANES given the uniform procedural standards for anthropometric measurements. Despite these limitations, the use of anthropometric measurements to estimate FFM and changes in FFM is simple, low cost, and has a predictive value of within 3 to 4 percent for 70 percent of the population [33,35] especially when adjusted for age. Finally, the unique strengths of the study include large sample size, standardized procedure for anthropometric measurements and its applicability to the entire United States population.

## Conclusion

In conclusion, we provide additional support for the differential effect of ethnicity on the loss of FFM and FFMI as the population ages. Regardless of the explanation for these differences, losses in muscle mass have several important health consequences, and perhaps more so for FFM decline. Most notable among these is increased functional dependency and mortality. This underscores the public health imperative of an ongoing epidemiological surveillance of the variations in FFM. If properly implemented, public health strategies may facilitate the design of targeted optimization of functional independency as the United States population ages.

## Abbreviations

Fat free mass (FFM); Fat free mass index (FFMI); Body mass index (BMI); The Third National Health and Nutrition Examination Survey (NHANES III)

## Competing interests

The author(s) declare that they have no competing interests.

## Authors' contributions

TOO conceived the study, participated in the design, performed statistical analysis and writing of the manuscript; MHA participated in the design, performed statistical analysis and writing of the manuscript; VB participated in the analysis and writing of the manuscript; RGA participated in conceiving and drafting of the manuscript, AA participated in the drafting of the manuscript, CNR participated in study design, statistical analyses and drafting of the manuscript

## Pre-publication history

The pre-publication history for this paper can be accessed here:


